# An explainable analysis of depression status and influencing factors among nursing students

**DOI:** 10.3389/fpsyt.2025.1696139

**Published:** 2025-12-02

**Authors:** Yingying Li, Bolun Sun, Xianzhe Wu, Yanchun Li

**Affiliations:** 1 School of Medicine, Qilu Institute of Technology, Jinan, Shandong, China; 2 Department of Pharmaceutical and Health Care Sciences, Jinan Technician College, Jinan, Shandong, China; 3 School of Nursing, Qilu Medical University, Zibo, Shandong, China

**Keywords:** nursing students, depression, extreme gradient boosting, XGBoost, nursing education

## Abstract

**Background:**

Depression is highly prevalent among nursing students (28.7%–30%). Although previous studies have identified multiple influencing factors, the lack of systematic prioritization hinders targeted intervention in resource-limited contexts. This study employed XGBoost and SHAP values to identify and prioritize key risk factors, thereby establishing a data-driven framework to assist educational administrators in optimizing resource allocation and facilitating early detection and personalized support.

**Methods:**

This multicenter cross-sectional study was conducted from September to December 2024 among nursing students recruited from ten universities in Shandong, Jiangxi, Henan, Hubei, and Sichuan provinces. Data were collected using a structured questionnaire comprising a demographic characteristics form, the Center for Epidemiological Studies Depression Scale (CES-D), and the Social Interaction Anxiety Scale (SIAS). Data cleaning was performed in Excel, and statistical analyses were conducted using SPSS Statistics version 27.0 and Python 3.9.

**Results:**

The incidence of depression among nursing students is 28.60%. According to the random forest model, the order of depression predicted by this study from high to low is Sleep Condition, Social anxiety, Mother's Educational Level, Sexual Orientation, Smoking, and Household composition.

**Conclusion:**

Depression is highly prevalent among nursing students, representing a significant challenge to both student well-being and the future healthcare workforce. This study identified and prioritized key determinants of depression, including poor sleep quality, social anxiety, low maternal education, sexual minority status, smoking, and single-parent family background. These findings can provide a basis for nursing administrators and educators to develop targeted and personalized intervention strategies.

## Introduction

A recent report by the World Health Organization highlights a worrying trend: around 14% of adolescents worldwide are affected by mental disorders, with depression being the leading cause of mental health conditions and health burden in this population (1).

This issue is particularly acute in higher education settings, where current data indicate that the prevalence of depression among college students is 17.3% (2), posing a serious challenge to these institutions (3). Furthermore, a high prevalence of depression is particularly evident among nursing students. Due to various stressors they encounter during their college years, they are more prone to depression (4, 5). The academic literature increasingly recognizes depression as a significant barrier to educational achievement in the university environment (6). Nursing students shoulder the heavy responsibility of saving the dead and healing the wounded (7). They are under pressure to acquire theoretical knowledge and professional skills (7). In addition, the pressures of clinical training experience represent a significant risk factor for psychological issues, with depression being the most common manifestation (8). Previous studies have confirmed that nursing students have a higher incidence of depression (9–11). A meta-analysis reported a pooled prevalence of depression among nursing students as high as 34.0%, with a notably higher prevalence of 43.0% in Asian subgroups (12). A study conducted in China reported a prevalence of 28.7% among nursing students (13). The detrimental impact of depression on nursing students' health extends to patient care, as it can hinder effective communication and patient engagement, thereby compromising care quality (11). Therefore, it is of great significance to identify the factors that affect nursing students' depression. While previous studies using traditional logistic regression have identified factors associated with depression in nursing students (14, 15), this method cannot quantify their relative importance or model complex interactions, limiting its utility for prioritizing interventions. To address this, our study employs the eXtreme Gradient Boosting (XGBoost) algorithm. XGBoost enhances predictive performance by integrating weak learners through structured loss function optimization and techniques like pre-sorting and weighted quantiles, which also prevent overfitting and improve generalization (16). Crucially, its built-in feature importance metrics provide an initial risk ranking. Furthermore, we integrate the SHapley Additive exPlanations (SHAP) framework to conduct an interpretability analysis. SHAP quantifies the direction and magnitude of each feature's impact on individual predictions (17), providing personalized insights. Therefore, by combining XGBoost's predictive power with SHAP's interpretability, this study aims to identify, prioritize, and interpret the key factors influencing depression among nursing students. The aim of this study is to provide nursing educators with a transparent, data-driven framework for the early identification of at-risk students and the formulation of targeted prevention strategies.

## Methods

### Participants

A multicenter cross-sectional study was conducted from September to December 2024 using a convenience sampling method. The study recruited 2,044 nursing students from ten universities across five provinces in China (Shandong, Jiangxi, Henan, Hubei, and Sichuan). Inclusion criteria were (1): enrollment as a nursing student and (2) provision of informed consent. The exclusion criterion was a clinical diagnosis of any mental health condition, regardless of medication status. We have supplemented the flowchart. For details, please refer to **Appendix Figure 1**.

### Data collection

The electronic questionnaire was distributed to potential participants through nursing faculty at each participating university.

### Ethical considerations

The study was conducted in accordance with the Declaration of Helsinki and was approved by the Ethics Committee of Qilu Institute of Technology (Approval No. QIT-2024-0081). Informed consent was obtained electronically from all participants before they could proceed to the survey questions. The survey was anonymous, and participation was voluntary.

### Sample size

The sample size was calculated based on the rule of thumb of having at least 10 events per variable (EPV) (18). With 19 variables anticipated for inclusion in the model, a minimum of 190 events was required. Considering an estimated depression prevalence of approximately 28.7% and a potential loss-to-follow-up rate of 20%, the initial target sample size for model development was calculated to be 238 participants. Furthermore, as the modeling sample typically constitutes 70% of the total dataset (with the remaining 30% reserved for validation), the total minimum sample size required was 340 participants. A total of 2,044 students completed the survey. After excluding respondents who answered in too short or too short, valid data were obtained for 2,024 participants, with an effective recovery rate of 99.022%.

### Measurement tools

#### Demographic characteristics

Based on a review of relevant literature (11, 19, 20) and consultations with field experts, we developed a demographic characteristics questionnaire. The questionnaire encompassed the following items: gender, age, ethnicity, educational level, school type, residence, household composition, household monthly income (¥), father's educational level, and mother's educational level.

#### Assessment of depression

The Center for Epidemiological Studies Depression Scale (CES-D), developed in 1977, is a widely used instrument for screening depressive symptoms (21). There are 20 items on the scale, graded 0-3, with a total score of 0–60 points. The assessment is conducted according to the actual situation in the latest week. No more than 1 day, 1–2 days, 3–4 days, and 5–7 days correspond to "no or almost no," "rarely," "often," and "almost always," respectively. The scale includes four dimensions: depressive mood, positive mood, physical disorder, and interpersonal relationship. A score of ≥16 indicates the presence of depressive symptoms (21). This scale has excellent reliability and validity (22, 23). In this study, the Cronbach’s α for the sample was 0.964.

#### Assessment of social anxiety

Social anxiety was evaluated using the Social Interaction Anxiety Scale (SIAS), compiled by Leary (24), the scale has a total of 15 items, and a single dimension is used to assess the tendency of subjective social anxiety experience independent of behavior. Using a 5-point scale, from "1" = "not at all" to "5" = "very consistent". The score ranges from 15 to 75 points, and 45 points are generally used as the standard for detecting social anxiety symptoms (25). In this study, the Cronbach’s α for the sample was 0.828.

### Statistical analysis

Data were analyzed by SPSS27.0 software. Count data were expressed as frequency and percentage. Univariate analysis was performed using the χ2 test. A P value < 0.05 was considered statistically significant. This study adopted Pandas, NumPy, and XG Boost libraries in the Python 3.9 environment for data entry, cleaning, transformation, and analysis. The dataset contains 2024 records with a total of 18 variables. All variables are of integer type (int64), and no missing values exist. During the modeling process, based on the variables extracted by single-factor analysis, the xg boost library was used to construct an Extreme Gradient Boosting Tree model to predict the degree of social fattening. This model is trained by integrating multiple gradient-boosting trees. The core hyperparameters include the maximum depth of the tree (max_depth), the number of trees (n_estimators), and the learning rate (learning_rate, with a default value of 0.1). The remaining parameters all use the default hyperparameters encapsulated in the xgboost library. To balance the computational cost of the model and the prediction performance, hyperparameter optimization is achieved through the Grid Search CV tool combined with 5-fold cross-validation, with specific ranges of max_depth (1 to 20) and n_estimators (5 to 50). Taking the Accuracy of the test set as the evaluation criterion, the optimal parameter combination is screened. Furthermore, the Shapley Additive Explanations (SHAP) method is utilized to conduct an interpretability analysis of the model. The optimal feature subset is screened by combining feature importance ranking and cross-validation to enhance the prediction accuracy and interpretability of the model.

## Results

### General characteristics of the participants

A total of 2,024 nursing students were investigated in this study, including 406 males and 1,618 females. Among them, 28.60% of the people were rated as depressed. **Table 1** shows the baseline characteristics of the depression group and the non-depression group. The Household composition, Household Monthly Income, Mother's Educational Level, Sleep Condition, Smoking, Alcohol drinking, Social anxiety, and Sexual Orientation of the two participant groups were evaluated, and the differences were statistically significant (P < 0.001).

**Table 1:** General characteristics of the participants.

Variables	Total	Depression		χ²	P
		No (N = 1445)	Yes (N = 579)		
Gender				3.793	0.051
Male	406	274(18.96%)	132 (22.80%)		
Female	1618	1171(81.04%)	447 (77.20%)		
Age(Years)				0.354	0.838
<20	1448	1029(71.21%)	419 (72.37%)		
20-23	553	400(27.68%)	153 (26.42%)		
≥24	23	16(1.11%)	7 (1.21%)		
Ethnic Group				0.001	0.997
Han Chinese	2003	1430(98.96%)	573 (98.96%)		
Ethnic Minority	21	15(1.04%)	6 (1.04%)		
Educational Level				0.070	0.792
Associate Degree or Below	1093	783(54.19%)	310(53.54%)		
Undergraduate Degree	931	662(45.81%)	269(46.46%)		
School type				0.076	0.782
Public School	962	684(47.34%)	278(48.01%)		
Private School	1062	761(52.66%)	301(51.99%)		
Clinical Practicum				2.693	0.101
Yes	752	892(61.73%)	380(65.63%)		
No	1272	553(38.27%)	199(34.37%)		
Residence				0.540	0.462
Urban Area	1148	827(57.23%)	321(55.44%)		
Rural Area	876	618(42.77%)	258(44.56%)		
Household composition				12.866	<0.001***
Two-parent family	1914	1383(95.71%)	531(91.71%)		
Single-parent family	110	62(4.29%)	48(8.29%)		
Household Monthly Income(¥)				7.339	0.025*
≥5000	641	483(33.46%)	158(27.29%)		
2000-4000	861	602(41.66%)	259(44.73%)		
<2000	522	360(24.91%)	162(27.97%)		
Father's Educational Level				1.217	0.544
Higher Education	307	219(15.16%)	88(15.20%)		
High School	455	334(23.11%)	121(20.90%)		
Junior High School or Below	1262	892(61.73%)	370(63.90%)		
Mother's Educational Level				9.616	0.008**
Higher Education	262	187(12.94%)	75(12.95%)		
High School	336	263(18.20%)	73(12.61%)		
Junior High School or Below	1426	995(68.86%)	431(74.44%)		
Singleton				2.199	0.138
Yes	466	320(22.15%)	146(25.22%)		
No	1558	1125(77.85%)	433(74.78%)		
Sleep Condition				136.418	<0.001***
Excellent	1222	968(66.99%)	254(43.87%)		
Fair	627	408(28.24%)	219(37.82%)		
Poor	175	69(4.77%)	106(18.31%)		
Smoking				18.559	<0.001***
Yes	75	37(2.56%)	38(6.56%)		
No	1949	1408(97.44%)	541(93.44%)		
Alcohol drinking				13.288	<0.001***
Yes	202	122(8.44%)	80(13.82%)		
No	1822	1323(91.56%)	499(86.18%)		
Religious Belief				1.664	0.197
Yes	128	85(5.88%)	43(7.43%)		
No	1896	1360(94.12%)	536(92.57%)		
Social anxiety					
Yes	521	308(21.31%)	213(36.79%)	51.772	<0.001***
No	1503	1137(78.69%)	366(63.21%)		
Sexual Orientation				14.798	<0.001***
Heterosexual	1878	1361(94.19%)	517(89.29%)		
sexual minority	146	84(5.81%)	62(10.71%)		
Academic Year				9.260	0.055
Secondary Vocational School	84	64(4.43%)	20(3.45%)		
Freshman	1082	765(52.94%)	317(54.75%)		
Sophomore	133	83(5.74%)	50(8.64%)		
Junior	248	176(12.18%)	72(12.44%)		
Senior	477	357(24.71%)	120(20.72%)		

*P<0.05, **P<0.01, ***P<0.001.

### Multivariate binary logistic regression analysis

Logistic regression analysis revealed that Mother's Educational Level (Higher Education) (OR = 0.718, P = 0.030, 95%CI:0.532 to 0.968), Sleep status (Excellent) (OR = 0.215, P<0.001, 95%CI: 0.152 to 0.305)) is a protective factor for depression among nursing students. Smoking (OR = 2.673, P < 0.001, 95%CI: 1.682 to 4.249), sexual minority (OR = 1.943, P <0.001, 95%CI: 1.378 to 2.739), single-parent family(OR = 2.016, P<0.001, 95%CI: 1.365 to 2.978) and social anxiety (OR = 2.148, P < 0.001, 95%CI: 1.740 to 2.652) are risk factors for depression among nursing students, as shown in **Table 2**.

**Table 2:** Multivariate binary logistic regression analysis of depression in nursing students.

Risk factor	Reference factor	B	SE	Waldx^2^	P	OR	95%CI
Mother's Educational Level	Junior High School or Below						
Higher Education		-0.332	0.152	4.731	0.030	0.718	0.532 to 0.968
Household composition	Two-parent family						
Single-parent family		0.701	0.199	12.430	<0.001	2.016	1.365 to 2.978
Sleep Condition	Poor						
Excellent		-1.535	0.177	75.021	<0.001	0.215	0.152 to 0.305
Fair		-0.890	0.181	24.043	<0.001	0.411	0.288 to 0.586
Smoking	No						
Yes		0.983	0.236	17.292	<0.001	2.673	1.682 to 4.249
Sexual Orientation	Heterosexual						
Sexual minority		0.664	0.175	14.371	<0.001	1.943	1.378 to 2.739
Social anxiety	No						
Yes		0.765	0.107	50.615	<0.001	2.148	1.740 to 2.652
Constant		2.587	0.390	44.014	<0.001	13.296	

### XG boost modeling results

#### Feature selection

Taking depression among nursing students (classified outcome) as the dependent variable, the regression analysis included Mother's Educational Level, Household composition, Sleep Condition, Smoking, Sexual Orientation and Social anxiety (P < 0.05) were included in the model.

#### Optimal subset

**Figure 1:**
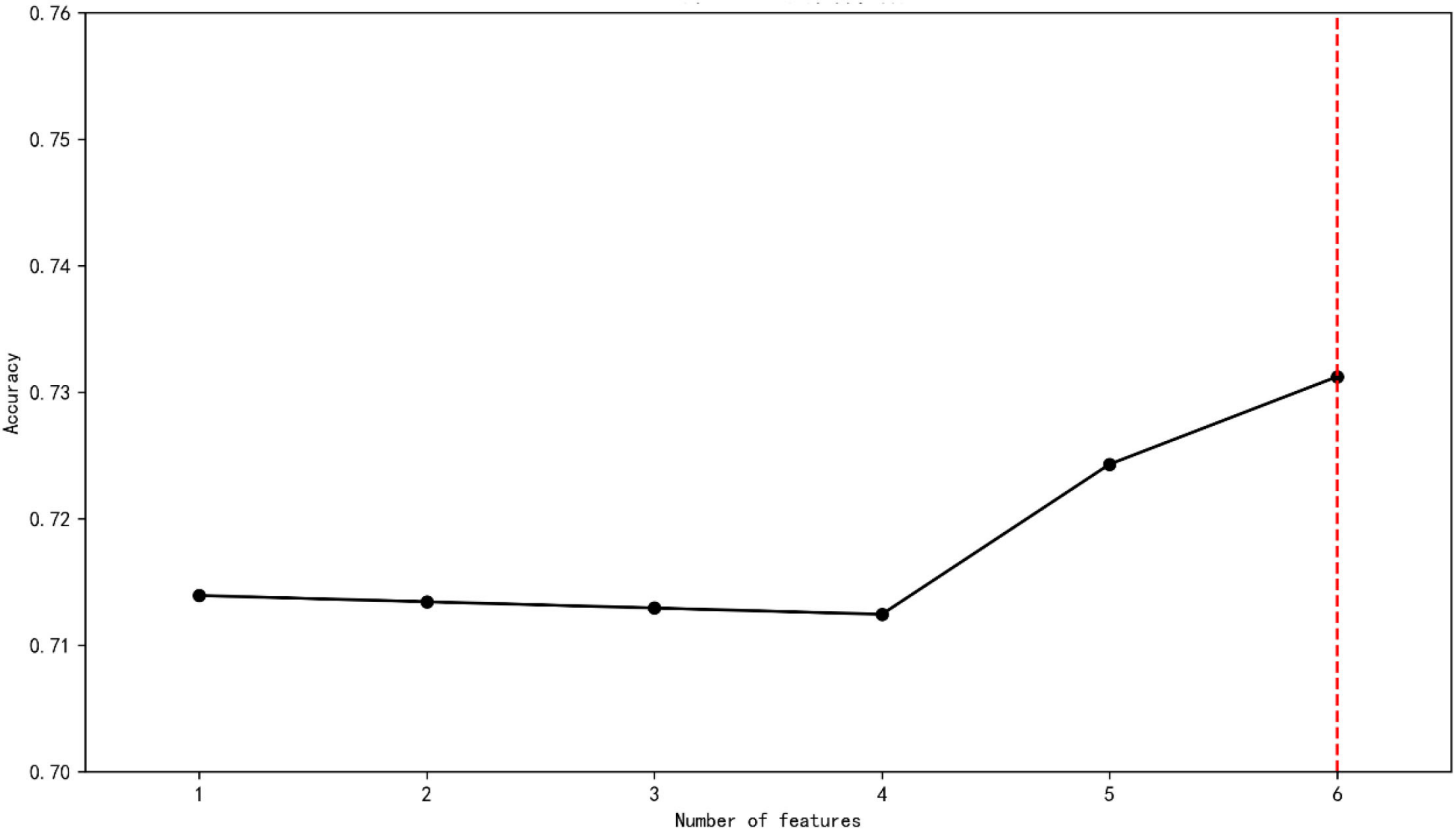
Screening the optimal feature subset.

To evaluate the model performance and screen the optimal feature subset(the features were derived from CES-D), 5-fold cross-validation (5-CV) is adopted to evaluate the classification accuracy under different numbers of features. The results show that when the number of features is 6, the cross-validation accuracy reaches the maximum value of 0.731 (see **Figure 1**).

### Analysis of model performance and SHAP interpretability

**Figure 2:**
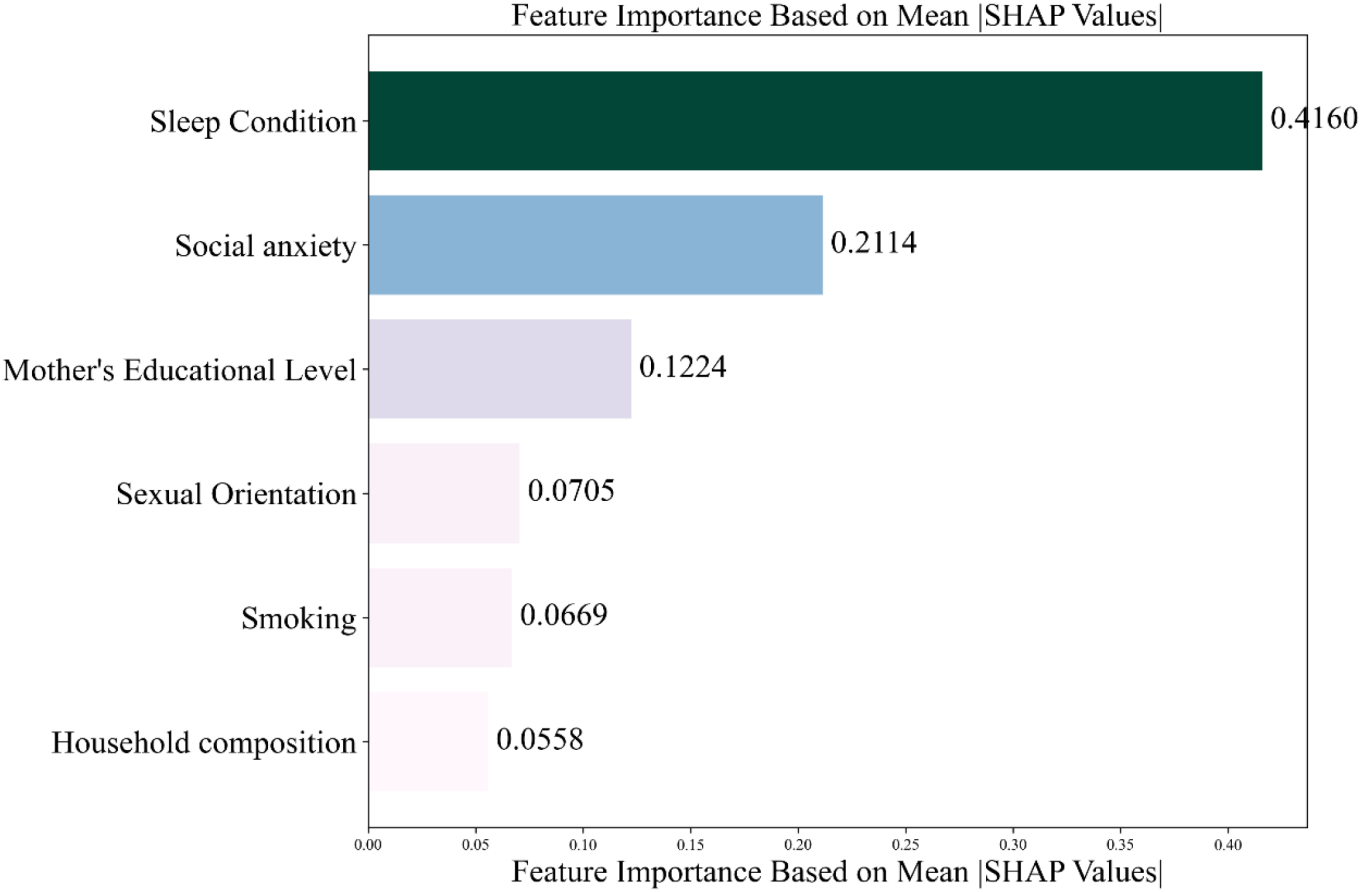
Feature importance ranking.

**Figure 3:**
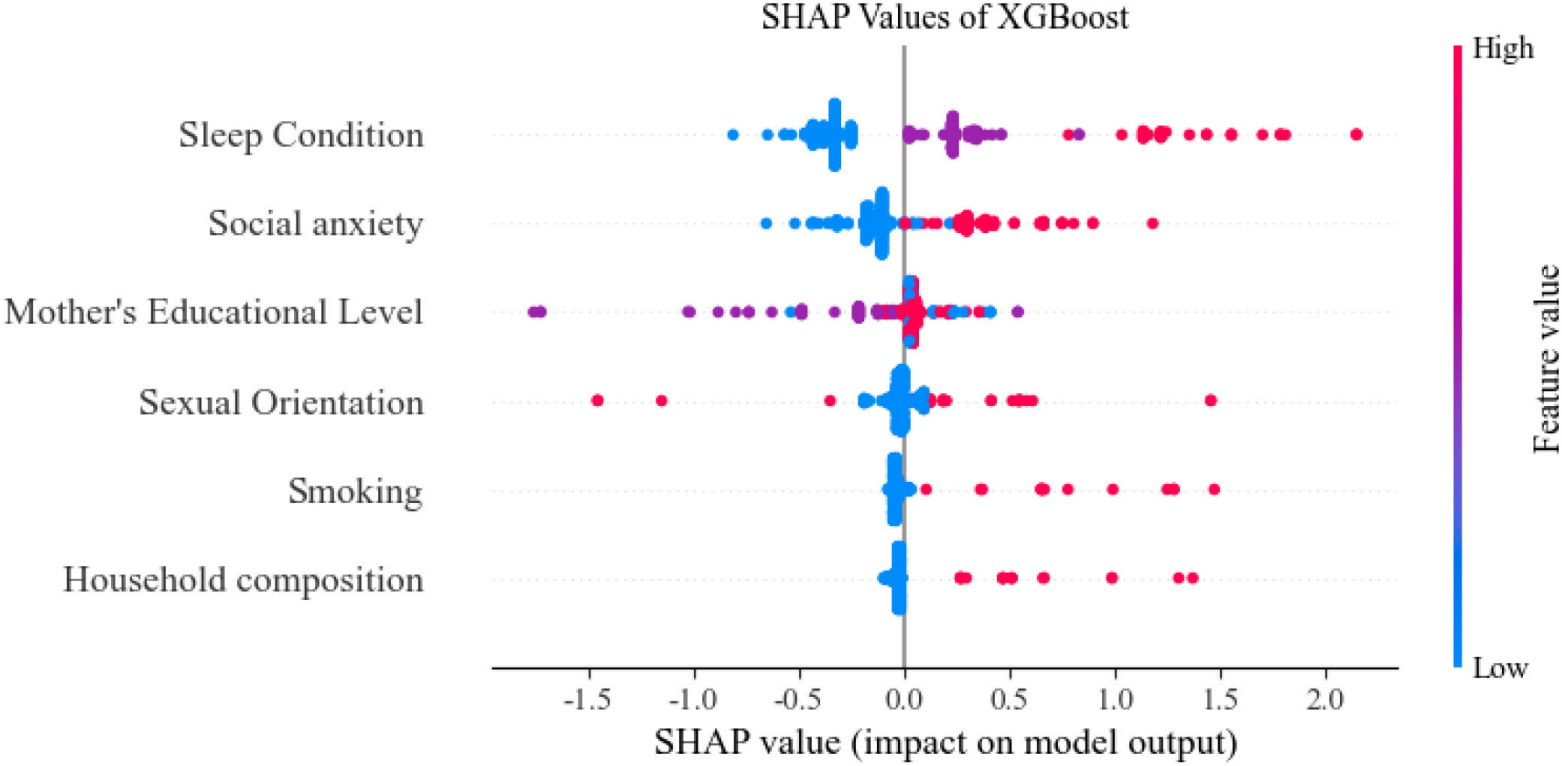
SHAP value (impact on model output).

Based on the optimal feature subset, the training set (1619 records) and the test set (405 records) were divided in an 8:2 ratio to construct the optimized XG Boost classification model. The optimal parameters are max_depth = 20 and n_estimators = 50. The accuracy rate of the training set is 0.7431 and that of the test set is 0.7580. SHAP analysis was used to reveal the contribution of characteristics to the prediction of depression among nursing students. The SHAP feature importance bar chart (see **Figure 2**) shows that the average absolute SHAP values from high to low((see **Figure 3**).

## Discussion

The study revealed a depression prevalence of 28.60% among nursing students. Univariate regression analysis, using a statistical significance threshold of P < 0.05, initially identified several factors associated with depression: mother’s educational level, household composition, sleep condition, smoking, sexual orientation, and social anxiety. To further quantify the relative influence of these predictors, an XGBoost model was trained and evaluated using built-in feature importance scores and SHAP values. The results indicated that the key factors, ranked in descending order of association with depression, were: sleep condition, social anxiety, mother’s educational level, sexual orientation, smoking, and household composition.

This study investigated 2,024 nursing students and found a depression prevalence of 28.60%. Nursing is a discipline with substantial theoretical and practical demands. Nursing students must undertake clinical practice, skill-based operations, and theoretical examinations, resulting in a heavier academic burden—a factor identified as increasing the risk of depression (12, 26). Furthermore, trainee nurses frequently interact with patients and their families in clinical settings and are required to respond to unexpected clinical situations with timely judgment and scientifically sound solutions. For those newly entering clinical practice, these demands can disrupt the learning process and contribute to the development of negative emotions (27).The observed prevalence rate of 28.60% in our study is lower than the 39.5% reported by Ji et al. (28)among medical students in Anhui Province, but higher than the 21.0% reported by Chen et al. (29) among university students in Wuhan. These disparities may be partially attributable to differences in study populations and regional contexts. Nevertheless, the relatively high prevalence of depressive symptoms among nursing students in our sample underscores the importance of early identification of relevant influencing factors and implementation of targeted interventions to reduce depression rates and promote psychological well-being in this population.

Household composition is an influential factor in the occurrence of depressive symptoms among nursing students, ranking second in importance in this study. Those from single-parent families are at a higher risk of developing depressive symptoms, which aligns with previous research findings (30–32). Family structure plays a significant role in adolescent development, and the family function and environment of nursing students can directly affect their psychological well-being (33). Studies have shown that adolescents from single-parent families often experience a lack of parental care, family support, and companionship. When facing challenges, they are more likely to feel isolated and helpless, and the lack of emotional support makes them reluctant to communicate about their problems. Prolonged exposure to such conditions can lead to the emergence of negative psychological behaviors such as depression and anxiety (34).

Multiple studies have indicated that individuals with diverse sexual orientations have a higher tendency to develop depression (35–37). One possible explanation may be rooted in socio-cultural contexts. Influenced by traditional values, same-sex relationships have not yet gained widespread acceptance in broader Chinese society, which may contribute to a climate of relatively low social recognition for diverse sexual orientations. Furthermore, these individuals may encounter limited understanding and acceptance from their families and communities. The persistent perception of inadequate support could adversely affect psychological adjustment, potentially predisposing individuals to depressive symptoms. Therefore, nursing students from single-parent families and those with same-sex partners require stronger social support and psychological resilience. Schools and families should provide greater care to reduce the incidence of depressive symptoms and improve their overall mental health.

Better sleep quality (rated as excellent or fair) showed a significant inverse association with depressive symptoms among nursing students in this cross-sectional analysis, which is consistent with the findings of Roberts (38)and Raudsepp (39). In our study, sleep conditions ranked first among all influencing factors. Research indicates that chronic sleep deprivation can disrupt the hypothalamic-pituitary-adrenal (HPA) axis, and dysregulation of this axis is associated with the development of depression (40). Furthermore, poor sleep quality may impair positive emotional response, mood regulation, short-term memory, and attention, while also reducing levels of brain-derived neurotrophic factor—thereby contributing to the onset of depression (41–43). Therefore, educational institutions should pay greater attention to the sleep conditions of nursing students and strive to provide a favorable sleep environment (44). In addition, nursing students themselves should also prioritize maintaining regular sleep patterns to enhance their mental health and reduce the risk of depression (44).

This study found that nursing students who smoke exhibit a higher risk of depressive symptoms, which aligns with previous research (45, 46). Mechanistically, long-term nicotine intake from smoking alters neurotransmitter activity in the brain, leading to impaired emotional regulation and deficits in memory and executive function (47, 48). Furthermore, smoking can disrupt the HPA axis, resulting in excessive cortisol secretion and subsequent neurobiological dysregulation, ultimately contributing to emotional disorders and negative affective states (49). Therefore, it is recommended that educational institutions and families provide tailored smoking cessation interventions for nursing students who smoke, along with training in emotional management and stress coping strategies, to mitigate depression risk. In this study, a significant inverse association was observed between mother's higher education and depression in nursing students. Higher maternal educational attainment is associated with more scientific parenting philosophies and enhanced mental health literacy. These attributes predispose individuals to adopt rational cognitive reappraisal and adaptive emotion regulation strategies (50), which collectively exert a positive influence on nursing students' psychological well-being and consequently reduce the incidence of depression (51). Family support serves as a crucial psychological resource in preventing and alleviating depression among nursing students. We recommend cultivating a positive and communicative family atmosphere, strengthening parent–child communication, attentively listening to academic and internship challenges, and providing constructive guidance rather than excessive criticism, which may otherwise exacerbate self-isolation and depressive symptoms (52).

Social anxiety was significantly associated with a higher prevalence of depressive symptoms among nursing students, a finding consistent with the research by Flynn et al. (53). Social anxiety is a common anxiety disorder primarily characterized by excessive worry and fear of negative evaluation by others in social or interpersonal contexts (54). Individuals with social anxiety tend to engage in negative self-evaluation during social interactions, leading to impaired emotion regulation ability (55). The mood-congruence effect also suggests that individuals in negative emotional states prioritize negative information. Thus, social anxiety can induce an attentional bias toward depressive emotional stimuli, thereby exacerbating the development of depression (56). It is recommended that nursing students engage in rational self-assessment, overcome feelings of inferiority and timidity, and acquire specific psychological coping strategies to enhance psychological resilience and promote mental health. Furthermore, nursing students should strengthen interpersonal communication skills, learn social techniques, improve team collaboration awareness, and establish positive interpersonal relationships. These measures can help reduce negative emotional experiences, maintain a positive mood, and prevent the occurrence of social anxiety.

The occurrence of depressive symptoms in nursing students can impair their cognitive functioning and empathic abilities, thereby compromising clinical judgment (14, 57, 58). This not only poses potential risks to patient safety but may also exacerbate the nursing workforce shortage (59). Furthermore, persistent depression can erode nursing students' professional identity and vocational commitment, thereby diminishing career satisfaction and impeding long-term professional development (58). The current nursing education environment, characterized by heavy academic pressure, a competitive job market, and frequent neglect of students' emotional needs, may inadvertently contribute to the development of depressive symptoms (60). Therefore, it is recommended that nursing educators and administrators implement systematic screening to comprehensively assess students’ mental health status and provide early interventions targeting identified risk factors.

## Strengths & limitations

To the best of our knowledge, this study represents the first application of the XGBoost algorithm to predict depression risk among nursing students. The use of XGBoost offers distinct advantages, including its ability to handle complex nonlinear relationships, mitigate overfitting through regularization, and provide interpretable feature importance rankings, thereby enhancing the robustness and clinical relevance of our predictive model. Furthermore, this multicenter, large-sample survey encompassing 2,024 nursing students improves the generalizability and statistical power of our findings compared to single-center studies. Several limitations of this study should be acknowledged. First, the cross-sectional design precludes causal inference among the variables. Future cohort or longitudinal studies are warranted to explore causal relationships and dynamic changes in depression among nursing students over time. Second, although predictor variables were selected based on a literature review, their scope may be limited, and important psychological or contextual factors could have been omitted. Additionally, the reliance on self-reported data may introduce recall or social desirability bias, and the geographical restriction of sampling may affect the extrapolation of results to other regions. Third, while this study ranked the importance of influencing factors, it did not explore potential interactions, mediating mechanisms, or specific pathways among variables. Although beyond the scope of this study, future research could address these aspects using methods such as path analysis or structural equation modeling. Nevertheless, future studies encountering more substantial missing data should employ more sophisticated methods, such as multiple imputation, to verify the robustness of their findings. The study utilized a convenience sample of nursing students from multiple universities. Due to the relatively homogeneous nature of this population, the generalizability of the findings to students of other majors or broader populations requires further verification. Finally, the results of our *post hoc* subgroup analyses are exploratory. Not corrected for multiple testing, and some subgroups may have had limited statistical power; therefore, these findings are hypothesis-generating and require cautious interpretation and external validation.

## Conclusion

This study adopted the XG Boost model to analyze the depression-related factors of 2024 nursing students. The key factors include Sleep Condition, Social anxiety, Mother's Educational Level, and Sexual Orientation Smoking. And Household composition. These research results indicate that nursing educators regularly screen for depression among nursing students, consider implementing supportive measures, provide psychological counseling promptly, improve the risk factors for the occurrence of depression among nursing students, and reduce the occurrence of depression. Future studies should explore the effectiveness of these intervention measures and investigate other factors influencing depression among nursing students.

## Data Availability

The original contributions presented in the study are included in the article/**Supplementary Material**. Further inquiries can be directed to the corresponding authors.
