# Supplementary figures and images for: An explainable analysis of depression status and influencing factors among nursing students
Source: Front Psychiatry. 2025 Dec 2;16:1696139. doi: 10.3389/fpsyt.2025.1696139 (PMC12705585; doi:10.3389/fpsyt.2025.1696139)

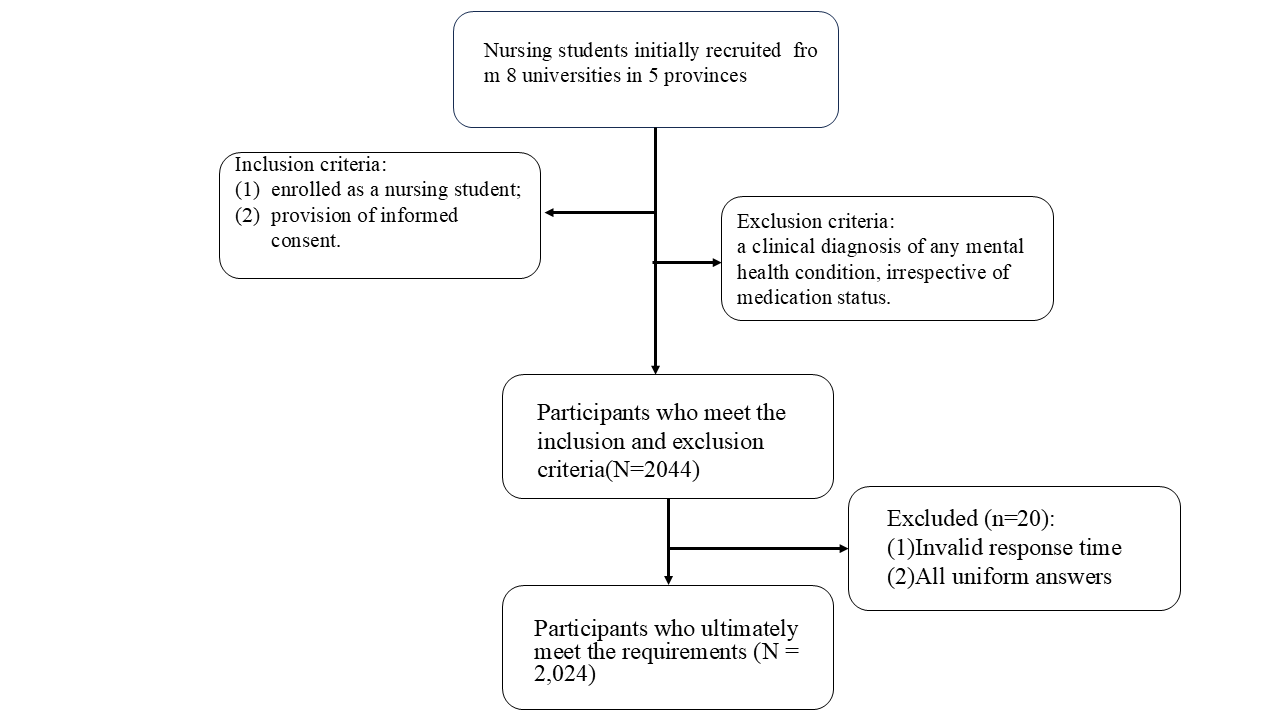

Supplement: Supplementary file 1 [file Image1.png]
